# Surfactant protein A promotes western diet-induced hepatic steatosis and fibrosis in mice

**DOI:** 10.1038/s41598-024-58291-5

**Published:** 2024-03-29

**Authors:** Ayobami Dare, Skylar D. King, Shi-You Chen

**Affiliations:** 1 Department of Surgery, University of Missouri School of Medicine, 1 Hospital Drive, Columbia, MO 65212 USA; 2 The Research Service, Harry S. Truman Memorial Veterans Hospital, Columbia, MO 65201 USA

**Keywords:** Physiology, Diseases, Medical research

## Abstract

Metabolic dysfunction-associated steatotic liver disease (MASLD) remains the most common cause of liver disease in the United States due to the increased incidence of metabolic dysfunction and obesity. Surfactant protein A (SPA) regulates macrophage function, strongly binds to lipids, and is implicated in renal and idiopathic pulmonary fibrosis (IPF). However, the role of SPA in lipid accumulation, inflammation, and hepatic fibrosis that characterize MASLD remains unknown. SPA deficient (SPA^−/−^) and age-matched wild-type (WT) control mice were fed a Western diet for 8 weeks to induce MASLD. Blood and liver samples were collected and used to analyze pathological features associated with MASLD. SPA expression was significantly upregulated in livers of mice with MASLD. SPA deficiency attenuated lipid accumulation along with downregulation of genes involved in fatty acid uptake and reduction of hepatic inflammation as evidenced by the diminished macrophage activation, decreased monocyte infiltration, and reduced production of inflammatory cytokines. Moreover, SPA^−/−^ inhibited stellate cell activation, collagen deposit, and liver fibrosis. These results highlight the novel role of SPA in promoting fatty acid uptake into hepatocytes, causing excessive lipid accumulation, inflammation, and fibrosis implicated in the pathogenesis of MASLD.

## Introduction

Metabolic dysfunction-associated steatotic liver disease (MASLD) has emerged as a healthcare burden globally due to the significant changes in dietary choices and lifestyle modifications^1,2^, and is a major cause of liver transplantation and hepatocellular carcinoma in the United States, with increased incidence across other populations because of the rising occurrence of insulin resistance, obesity, and diabetes^3,4^. MASLD is a progressive liver disease that ranges from simple liver steatosis to steatohepatitis, characterized by inflammation, necrosis, and fibrosis, which can eventually progress to cirrhosis and/or hepatocellular carcinoma^5^. An imbalance in nutrient consumption to energy ratio contributes majorly to the etiology of MASLD^6^. Excessive lipid accumulation can initiate and aggravate hepatocyte injury as well as facilitate other pathogenic mechanisms including macrophage infiltration and stellate cell activation that may worsen the liver injury, thereby promoting the progression of MASLD^7,8^. The underlying mechanisms regulating hepatic lipid homeostasis are heterogeneous, including de novo lipogenesis, lipid uptake, fatty acid oxidation, lipid export, and other biochemical signals^9^. Therefore, evaluating the specific signals underlying the metabolic derangement of lipids and its associated inflammatory responses remains an active area of research interest, with the principal goal of improving MASLD management.

Surfactant protein A (SPA), a protein secreted by alveola type-II cells, is expressed in pulmonary and extrapulmonary tissues, including skin, digestive tract, tear ducts, and salivary glands^10–12^. SPA is a 28–36 kDa glycoprotein belonging to the collectin family, comprising of a C-terminal carbohydrate recognition domain, a collagen domain, and a NH_2_-terminal domain rich in cysteine. SPA functions as a surface tension-reducing agent and regulates immune homeostasis^13,14^. Although mild alteration in surfactant metabolism was reported in SPA-deficient mice, no significant changes in pulmonary function were observed compared to wild-type^15,16^, suggesting that SPA-deficient mice may be used as a model to evaluate the extrapulmonary functions of SPA.

Previous studies have shown that SPA specifically and strongly binds to lipids, which is dependent upon calcium ions and the integrity of its collagen domain^17^. SPA contributes to atherosclerosis by promoting foam cell formation in macrophages and upregulating CD36 expression^18^. SPA can bind to cell surface receptors such as calreticulin and activate MAPK/NF-kB signaling cascade with a resultant increase in downstream inflammatory mediators that promote the development/progression of renal fibrosis^19^. Also, a variant in human surfactant protein A1 has been reported to enhance pulmonary epithelial cell secretion of TGF-β1, resulting in lung fibrosis^20^. Additionally, the elevated serum level of SPA has been identified as a potent biomarker of lung disease and a unique predictor of early mortality among idiopathic pulmonary fibrotic patients^21,22^. Since lipid accumulation and hepatic inflammation are underlying pathological processes in MASLD, we hypothesized that SPA promotes the development of MASLD induced by western diet (W-D). Indeed, we found that SPA promotes fatty acid uptake, hepatic inflammation, and stellate cell activation in the livers of mice fed W-D. This is the first study showing a significant contribution of SPA to liver steatosis and fibrosis.

## Materials and methods

### Animals

SPA deficient (SPA^−/−^) mice (sftpa 1^tm1kor^/J, stock no: 004964) were purchased from the Jackson Laboratory and have been crossbred with C57BL/6 for more than ten generations. The age-matched wildtype littermates were used as controls. All animals were housed under conventional laboratory conditions with humane care following the principles of Animal Care formulated by the National Society for Medical Research and guidelines for the use of laboratory animals. All animal procedures were approved by the Animal Care and Use Committee of the University of Missouri. Male mice were used in this study to evaluate the contribution of SPA in liver steatosis and fibrosis due to their increased susceptibility to fatty diet^23^. Future study will include female mice to examine if gender is a confounding variable in the role of SPA in MASLD. SPA^−/−^ and age-matched wild type (WT) animals were randomly distributed into two groups (n = 7) and fed with normal chow or western diet (D19061310i, Research diets; 40 kcal% Fat (Mostly Palm Oil), 20 kcal% Fructose, 2% Cholesterol and 0.5 g Choline) for 8 weeks. Body weight was monitored every week. At the end of the experiment, blood samples were collected from anesthetized animals. The livers were excised, cleared of adherent tissues, and weighed. The portions of liver tissues were embedded in optimum cutting temperature (OCT) compound for Oil-Red O staining or fixed in 4% buffered formalin phosphate, while the remaining livers were kept at − 80 °C for further analysis.

### Biochemical analysis

Blood samples were kept at room temperature for 20 min and centrifuged at 3000 rpm at 4 °C for 15 min using a Beckman Coulter centrifuge to obtain serum. 50 µL of serum were sent to MU Veterinary Medical Diagnostic Laboratory for analysis of serum triglycerides (TG), cholesterol (TC), alkaline phosphatase (ALP), alanine transaminase (ALT) and aspartate transferase (AST) using AU480 clinical Chemistry Analyzer. Liver homogenates were obtained as described^24^. Briefly, 200 mg of liver tissue was homogenized in 1 mL of PBS containing 0.5% Triton-X. The homogenates were centrifuged at 10,000 rpm and 4 °C for 10 min to separate the supernatant. Liver triglyceride and cholesterol contents in the supernatant were measured using the same AU480 Clinical Chemistry Analyzer.

### Quantitative polymerase chain reaction (qRT-PCR)

Total RNA from liver tissues (50–100 mg) were extracted using Trizol (1 mL) reagent (Thermo Scientific, 10296028). The RNA concentration was determined by measuring absorbance at 260/280 nm with a Nanodrop One spectrophotometer (Thermo Scientific). The integrity of the extracted RNA was verified using gel electrophoresis to assess the 28S and 18S band ratios. The RNA was converted to cDNA by reverse transcription using iScript cDNA Synthesis Kit (Bio-Rad, 1708890). qRT-PCR was conducted using SYBR Green master mix (GeneCopoeia, QP001) in QuantStudio-3, an Applied Biosystems PCR instrument. Relative mRNA expression of the genes of interest was calculated using the 2^−ΔΔct^ method^25^ and normalized to cyclophilin (CYP, the endogenous control). The primers used in this study are listed in Table 1, and their specificity were verified by using the NCBI BLAST tool.

**Table 1:** Primers used in this study.

Genes	Primer sequence
PPARγ	F: 5′-CATTCTGGCCCACCAACTTCG-3′ R: 5′-CACTGGCCTTGGTGGAAGAT-3′
CD36	F: 5′-TGAATGGTTGAGACCCCGTG-3′ R: 5′-TACGTGGCCCGGTTCTACTA-3′
FASn	F: 5′-GGACACACAGCATTAGGGACAT-3′ R: 5′-TGGGATGCTTGATCTGCTGTA-3′
SCD1	F: 5′-CTACAAGCCTGGCCTCCTGC-3′ R: 5′-GGACCCCAGGGAAACCAGGA-3′
PPARα	F: 5′-TGCAGCCTCAGCCAAGTTGAA-3′ R: 5′-AGCCACAACGTTTCACA-3′
NFkB	F: 5′-AGGCTTCTGGGCCTTATGTG-3′ R: 5′-TGCTTCTCTCGCCAGGAATAC-3′
CCl2	F: 5′-AACTGCATCTGCCCTAAGGT-3′ R: 5′-AGGCATCACAGTCCGAGTCA-3′
TNFα	F: 5′-ACAGAAAGCATGATCCGCGA-3′ R: 5′-GTTTGCTACGACGTGGGCT-3′
IL-1β	F: 5′-GAAATGCCACCTTTTGACAGTG-3′ R: 5′-CTGGATGCTCTCATCAGGACA-3′
Acta2	F: 5′-GGCTCTGGGCTCTGTAAGG-3′ R: 5′-CTCTTGCTCTGGGCTTCATC-3′
TGFβ1	F: 5′-GTCACTGGAGTTGTACGGCA-3′ R: 5′-AGCCCTGTATTCCGTCTCCT-3′
Col1a1	F: 5′-GCTCCTCTTAGGGGCCACT-3′ R: 5′-CCACGTCTCACCATTGGGG-3′
CYP	F: 5′-GTGGTCTTTGGGAAGGTGAA-3′ R: 5′-TTACAGGACATTGCGAGCAG-3′
CPT1a	F: 5′-CTCCGCCTGAGCCATGAAG-3′ R: 5′-CACCAGTGATGATGCCATTCT-3′

### Western blotting

Total proteins were extracted from freshly isolated liver tissue (5 mg) and lysed in 1 × RIPA lysis buffer (EMD Millipore, #20-188) containing 1% protease and phosphatase inhibitors (Thermo Scientific). Protein concentrations were measured using Pierce BCA protein assay kit (#23227) by following the manufacturer’s recommendations. 20 µg of protein were dissolved on SDS–polyacrylamide gels, transferred onto a PVDF membrane (Bio-Rad), and blocked with 5% Bovine Serum Albumin for 1 h at room temperature. The membrane was incubated with primary antibodies (1:1000) at 4 °C overnight and IRDye secondary antibodies (LI-COR Biosciences) at room temperature for 1 h. Protein expression was detected and quantified by Odyssey CLx Imaging System (LI-COR Biosciences). Polyclonal mouse primary antibody for SPA (1:100) was made as previously reported^18^, VCAM1 (#6294-1-Ig), and GAPDH (60004-1-Ig) were used for immunoblotting.

### Immunofluorescent staining

Tissues were fixed in buffered formalin phosphate and embedded in paraffin. Liver sections (5 µm) were heated, deparaffinized in xylene, rehydrated, and underwent antigen-retrieval in Tris–EDTA buffer (100 mM Trisbase, 1 mM EDTA, 0.05% Tween-20, pH 9.0). Sections were then washed in PBS containing 0.1% Tween-20, blocked in 10% goat serum at room temperature for 1 h, and then incubated with primary antibodies against SPA (1:100), ACTA2 (Sigma #A2547, 1:100); F4/80 (Abcam, #ab16911, 1:100) at 4 °C overnight. In the following day, sections were incubated with fluorescent dye-conjugated secondary antibody (1:100) at room temperature for 1 h and mounted with cover slides using a medium containing DAPI (Vectashield antifade mounting media, Vector laboratories). Images were captured with a Keyence BZ-X810 or a Leica S8 confocal microscope and processed using ImageJ.

### Tissue histology

Liver tissues were fixed in buffered formalin phosphate and embedded in paraffin. 5 µm sections mounted on a slide were stained with Hematoxylin and eosin (H&E) for histological analysis. Stained liver sections were reviewed in a blinded fashion by a pathologists who scored the severity of hepatic steatosis and lobular inflammation using the method reported by^26^, and hepatocyte ballooning was scored using the method reported by^27^. To evaluate lipid deposition, liver sections were stained with Oil Red-O (ORO). Picrosirius Red staining was used to evaluate collagen deposition and liver fibrosis. Images were captured under the microscope. Lipid deposits (i.e., the percentage of Oil Red-O-stained area) and fibrotic area (i.e., the collagen deposition) were quantified using Image-J analysis software.

### Statistical analysis

All data represent independent points and are present as mean ± standard error (SE). The normality test was performed using the Shapiro–Wilk test. Student's unpaired t-tests were used to compare two groups, while a one-way ANOVA with Tukey post-hoc test was used for more than two groups. Statistical analyses were conducted using GraphPad Prism 9.0 (GraphPad Software, CA), and p < 0.05 was considered significant. This study is reported in accordance with ARRIVE guidelines.

## Results

### SPA expression is upregulated in livers of mice fed western diet

**Figure 1:**
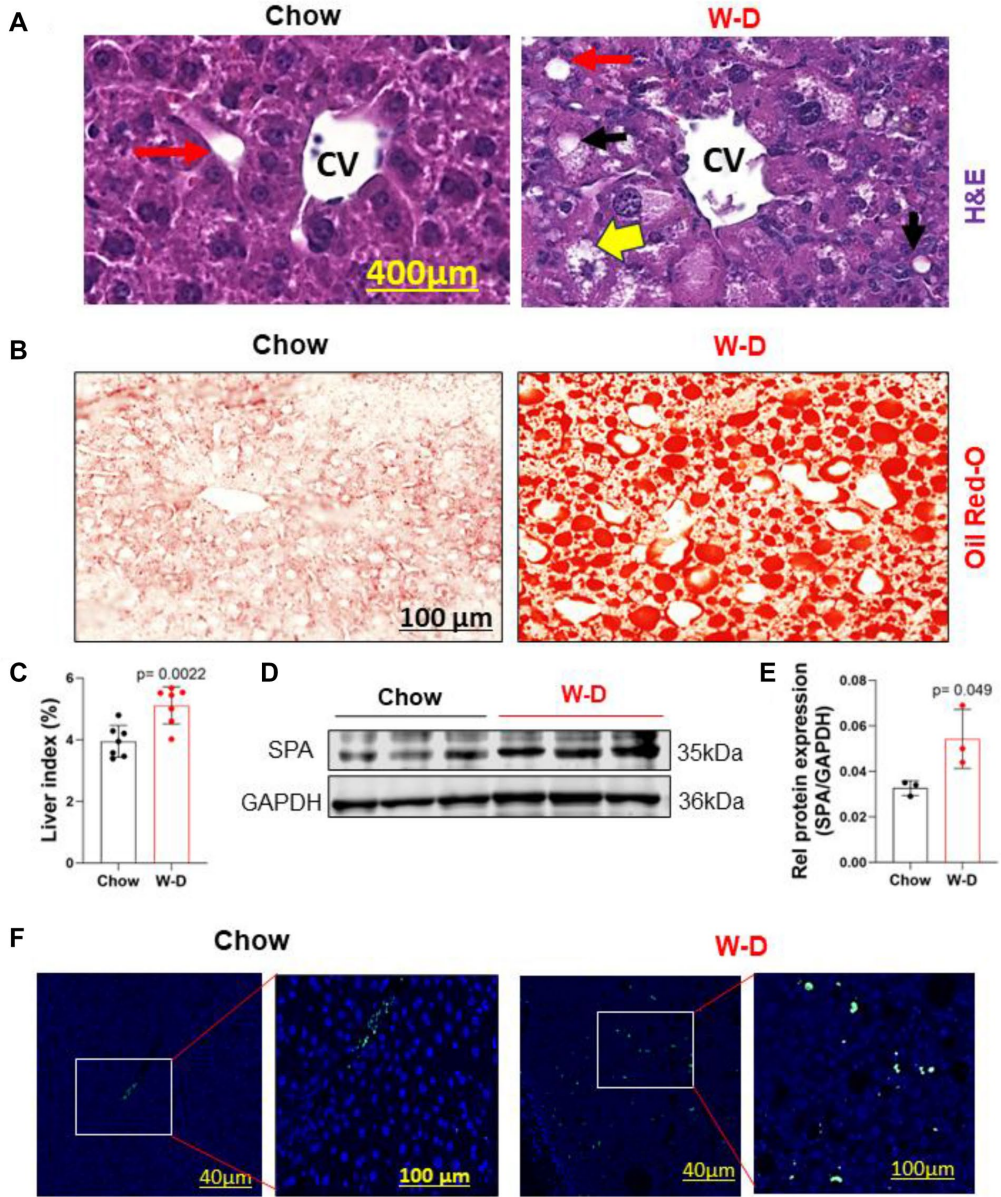
SPA was increased in liver tissues of mice with a western diet. Male mice fed a Chow or Western diet (W-D) for 8 weeks. (**A**) H&E staining of liver sections (CV—Central vein; red arrow—portal vein; yellow arrow—hepatocyte degeneration; black arrow—fat deposits). (**B**) Oil Red-O staining of liver sections. (**C**) Liver index shown as percentage of liver/body weight. (**D**) Hepatic SPA expression detected by Western blot. (**E**) Quantification of hepatic SPA protein expression shown in (**D**) by normalizing to GAPDH. (**F**) Immunofluorescent staining of SPA (green) in liver tissues. Nuclei was stained with DAPI (blue).

Mice fed a W-D for 8 weeks show steatohepatitis in their livers^28,29^. H&E and Oil Red-O staining of liver sections in Chow diet-fed mice showed normal liver cytoarchitecture with no lipid staining; however, the liver sections from W-D fed mice displayed significant lipid accumulation (red stains) and hepatocytes degeneration (yellow arrows) (Fig. 1A,B). Liver index, shown as a percentage of liver/body weight, significantly increased in the W-D fed mice compared to Chow diet fed mice (Fig. 1C). Importantly, SPA protein expression was significantly upregulated in livers of mice with W-D as shown by Western blot analyses (Fig. 1D,E), which was also confirmed by immunofluorescent staining (Fig. 1F).

### SPA deficiency attenuates steatosis and liver injury due to western diet feeding

**Figure 2:**
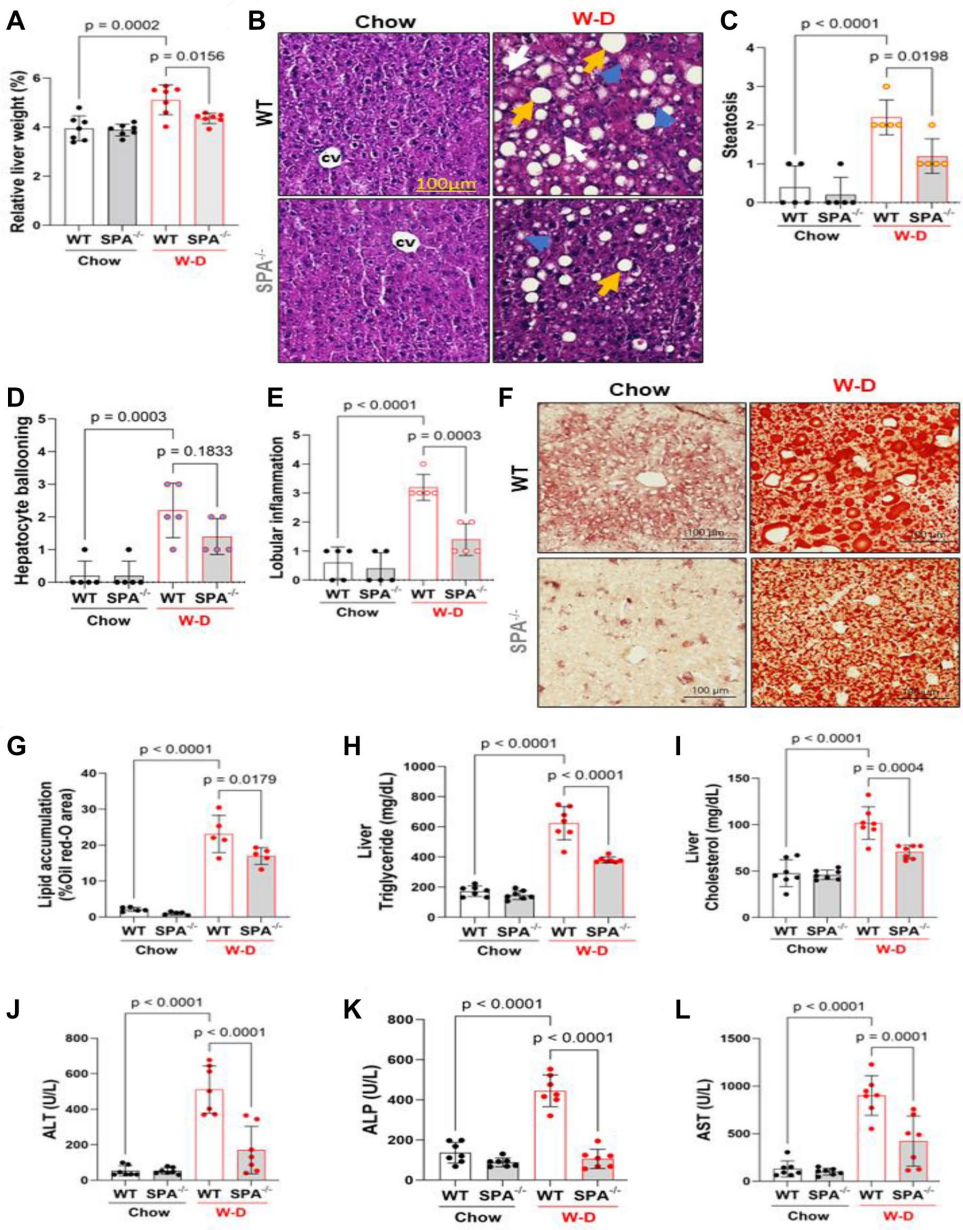
Western diet (W-D)-induced steatosis and liver injury were reduced in SPA deficient mice. WT or SPA^−/−^ mice fed Chow or W-D for 8 weeks. (**A**) Relative liver weight showing as percentage of liver/body weight ratios in WT or SPA^−/−^ mice fed Chow or W-D for 8 weeks. (**B**) H&E staining of liver sections. Arrows indicate steatosis (yellow), hepatocyte ballooning (blue), and inflammatory cells (white). (**C**) Quantification of **s**teatosis (0: < 5% steatosis, 1: 5–33% steatosis, 2: 33–66% steatosis, 3: > 66% steatosis). (**D**) Quantification of hepatocyte ballooning (0: no ballooning, 1: only non-classic ballooning, 2: few classic ballooning, 3: many classic ballooning, 4: severe classic ballooning). (**E**) Quantification of inflammatory cells (0: no foci, 1: < 2 foci per ×200 field, 2: 2–4 foci per ×200 field, 3: > 4 foci per ×200 field) (**F**) Oil Red-O staining of liver sections. (**G**) Quantification of lipid accumulation showing as percentage oil Red-O stain relative to the total section areas. (**H**) Triglyceride levels. (**I**) Cholesterol contents in the livers. (**J**) Serum concentrations of alanine transaminase. (**K**) Serum alkaline phosphatase (ALP) levels. (**L**) Serum aspartate transferase (AST).

To evaluate the role of SPA in hepatic steatosis and associated liver injury, H&E and Oil Red O-staining and biochemical analyses of liver sections of SPA^−/−^ and WT mice fed Chow diet or W-D were conducted. Significantly increased liver/body weight ratios (Fig. 2A), lipid accumulation, hepatocyte ballooning, and lobular inflammation (Fig. 2B–G) were observed in the livers of WT mice fed W-D relative to WT mice fed a Chow diet as previously reported^29^. Additionally, we found elevated triglycerides and total cholesterol in the livers (Fig. 2H,I). Serum concentrations of liver enzymes, including ALT, ALP, and AST were also significantly increased in WT mice fed W-D compared to the Chow diet (Fig. 2J–L). However, SPA^−/−^ mice fed W-D exhibited significantly reduced relative liver/body weight ratios, lipid accumulation, hepatocyte ballooning, lobular inflammation, liver contents of triglycerides and cholesterol, and serum levels of liver enzymes as compared to the W-D fed WT mice. These data indicated that SPA is involved in lipid accumulation and liver injury in mice with W-D (Supplementary Figure 1).

### SPA deficiency reduces liver fatty acid uptake

**Figure 3:**
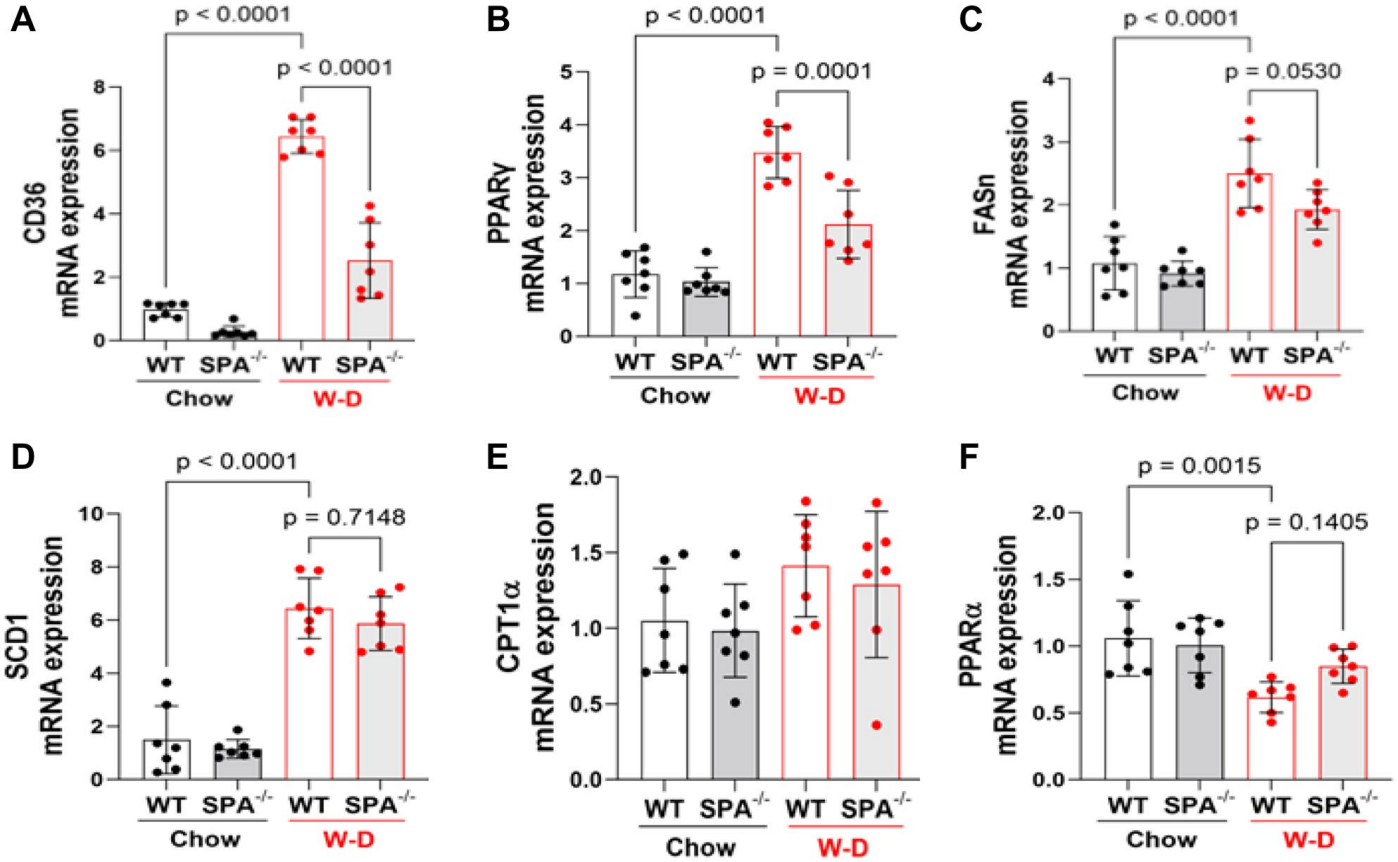
Fatty acid uptake was reduced in western-diet fed SPA^−/−^ mice. Relative mRNA expression of genes regulating fatty acid homeostasis was detected by qRT-PCR and normalized to cyclophilin expression in the liver of WT or SPA^−/−^ mice fed Chow or W-D for 8 weeks. (**A**) Cluster of differentiation 36 (CD36). (**B**) Peroxisome Proliferator-Activated Receptor-γ (PPAR-γ). (**C**) Fatty Acid Synthase (FASn). (**D**) Stearoyl-CoA desaturase 1 (SCD1). (**E**) Carnitine Palmitoyltransferase-1 (CPT1). (**F**) Peroxisome Proliferator Activated Receptor-γ (PPAR-α).

In order to understand how SPA promotes the liver steatosis, we analyzed the mRNA expression of genes involved in fatty acid uptake, lipogenesis, and oxidation. SPA^−/−^ significantly reduced the mRNA expression of genes regulating fatty acid uptake such as peroxisome proliferating activator receptor-gamma (PPARγ) and cluster of differentiation 36 (CD36) in mice fed W-D compared with WT mice fed W-D (Fig. 3A,B). However, SPA deficiency did not alter the mRNA expression of lipogenic genes, fatty acid synthase (FASn) and stearoyl-CoA desaturase 1 (SCD1) or genes regulating fatty acid oxidation, i.e., carnitine palmitoyltransferase-1 (CPT1) and peroxisome proliferator activated receptor-γ (PPAR-α) in mice fed W-D (Fig. 3C–F). These data indicate that SPA promotes liver steatosis by stimulating liver fatty acid uptake.

### SPA deficiency reduces liver inflammation in western-diet-induced MASLD

**Figure 4:**
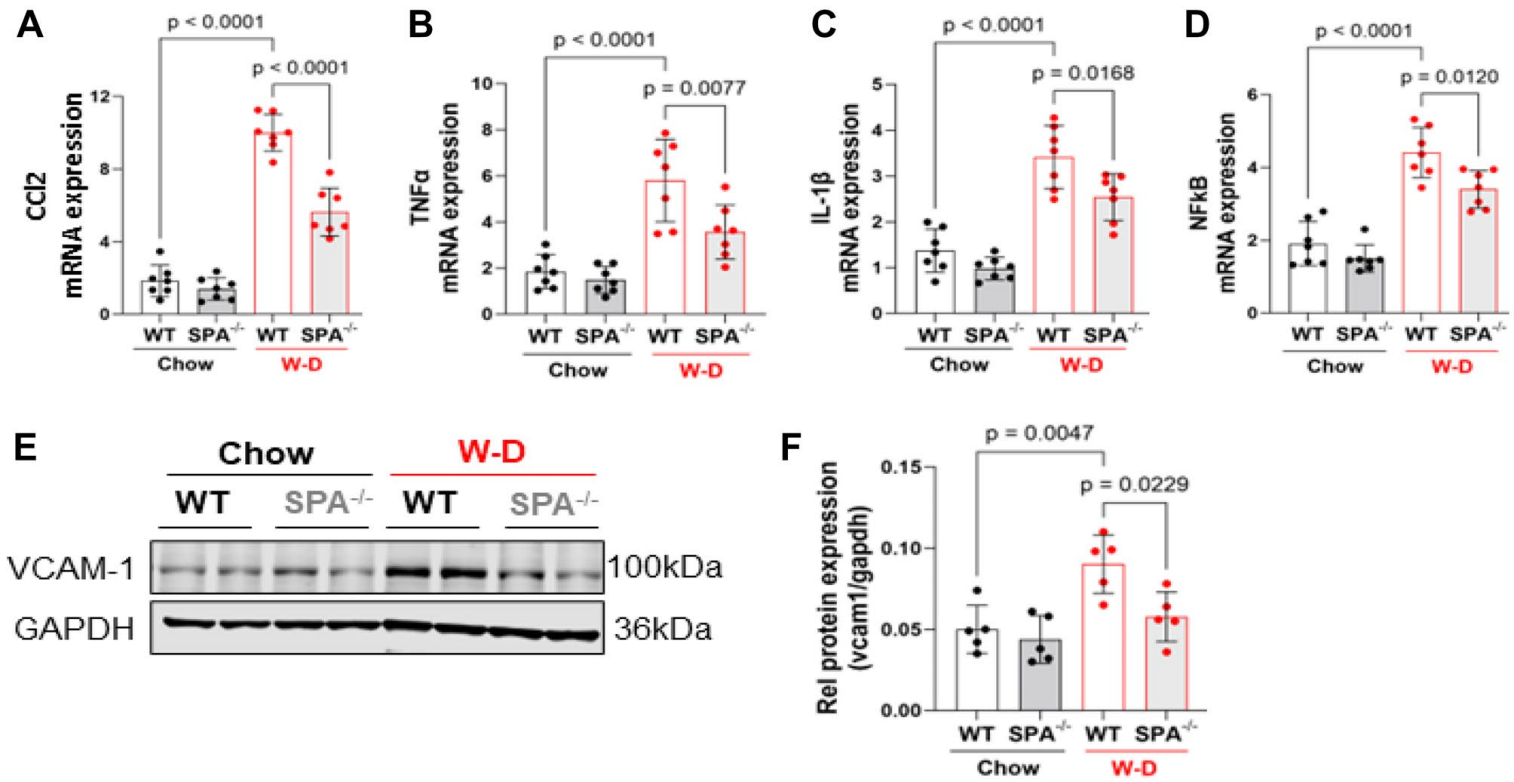
SPA deficiency attenuated western diet-induced liver inflammation. WT or SPA^−/−^ mice were fed Chow or W-D for 8 weeks. The expression of pro-inflammatory genes in livers was detected by qRT-PCR, and the fold change of each mRNA level was normalized to the cyclophilin expression. (**A**) Chemokine (C–C motif) Ligand-2 (CCl2). (**B**) Tumor Necrotic Factor-α (TNF-α). (**C**) Interleukin-1 beta (IL-1β). (**D**) Nuclear Factor Kappa-B (NF-kB). (**E**) Vascular cell adhesion molecule-1 (VCAM-1) expression detected by Western blot. (**F**) Quantification of VCAM-1 protein expression shown in D by normalized to GAPDH.

Immune cell infiltration into the liver and macrophage activation play important roles in the initiation and development of liver inflammation during steatohepatitis^30^. It is well known that nuclear factor kappa-B (NF-kB) signaling pathway is critical in inflammatory response and has also been implicated in MASLD^31^. Thus, we evaluated the role of SPA in W-D-induced hepatic inflammation. A significant decrease in the expression of NF-kB and its downstream chemokine (C–C motif) ligand-2 (CCl2) and cytokines tumor necrotic factor-alpha (TNF-α) and Interleukin-1beta (IL-1β) was observed in SPA^−/−^ mice fed W-D as compared with WT mice fed W-D (Fig. 4A–D), which is consistent with previous studies showing that SPA promotes pro-inflammatory cytokine and chemokine production via activating NF-kB signaling^32^. It appears that SPA NH2-terminal binds to macrophage calreticulin/CD91 to activate NF-kB and generate proinflammatory cytokines including TNF-α^33^. Moreover, the protein expression of vascular cell adhesion molecule-1 (VCAM1) was reduced in SPA^−/−^ mice fed W-D compared with WT mice (Fig. 4E,F). VCAM1 is a vascular cell surface protein that induces adherence and extravasation of monocytes to blood vessels and links lipotoxicity with endothelial dysfunction^34^.

### SPA deficiency attenuates stellate cell activation and hepatic fibrosis in western diet-induced MASLD

**Figure 5:**
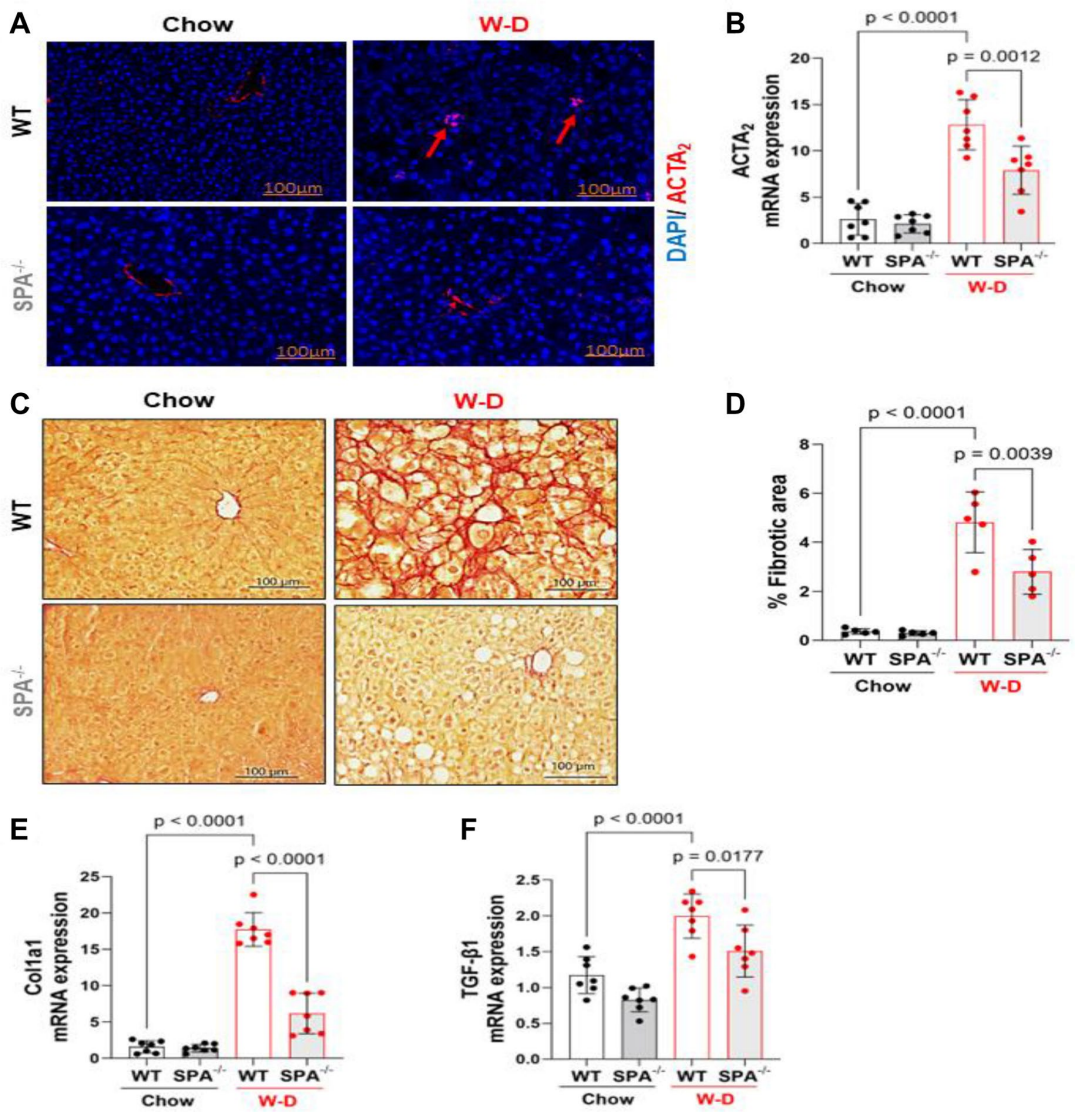
SPA deficiency attenuated stellate cell activation and hepatic fibrosis in western-diet fed mice. (**A**) Immunostaining of actin alpha-2 (ACTA_2_) in the liver from WT or SPA^−/−^ mice fed Chow or W-D for 8 weeks. (**B**) mRNA expression level of ACTA2 in the livers. (**C**) Picro Sirius Red staining for collagen deposition in liver tissues. Collagen deposit is indicated by the red stain. (**D**) Quantification of fibrotic area showing as the percentage of the total area in the tissue (n = 5). (**E**) Collagen type alpha 1 (Col1a1) mRNA levels. (**F**) transforming Growth factor-β1 (TGF-β1) mRNA levels.

Stellate cell activation and hepatic fibrosis are critical process in the progression of MASLD. We thus evaluated the role of SPA in stellate cell activation and fibrosis in mouse livers after 8 weeks of W-D feeding. As shown in Fig. 5A, strong actin alpha-2 (ACTA2) staining, a unique marker of stellate cell activation, was observed in multiple liver cells from WT mice fed W-D. However, the ACTA2^+^ cell numbers were significantly reduced in SPA^−/−^ mice fed W-D. Consistent with the ACTA2 immunostaining, SPA^−/−^ mice fed W-D exhibited a significant reduction in liver mRNA expression of ACTA2 compared to WT mice fed W-D (Fig. 5B). Furthermore, the Picro Sirius red staining showed a significant decrease in collagen deposition in the livers of SPA^−/−^ mice fed a W-D as compared to the excessive collagen deposition observed in the WT mice fed W-D (Fig. 5C,D). Furthermore, the expression of collagen type alpha 1 (Col1α1) and transforming growth factor-beta 1 (TGF-β1) was also significantly reduced in the livers of SPA^−/−^ mice fed W-D compared to WT mice fed W-D (Fig. 5E,F). These results indicate that SPA^−/−^ may inhibit stellate cell activation and further liver fibrosis.

## Discussion

Several studies have demonstrated that a therapeutic regimen directed at lipid homeostasis improves liver function in alcoholic and nonalcoholic fatty liver disease^35,36^. The mouse model of MASLD used in this study has been reported to show a similar spectrum of liver complications to human MASLD, including hepatic lipid accumulation, inflammation, liver injury, and fibrosis^28,29^. Thereby, the model provides a platform to evaluate the contribution of SPA in the pathogenesis of MASLD. In this study, we establish that SPA significantly promotes the pathological characteristics of MASLD due to our observations in the SPA-deficient mice fed a W-D.

Previous studies have reported an increased serum SPA level in obese and type-2 diabetic rats with extensive lipid deposition^37,38^. We observed that both protein and cellular expression of SPA are upregulated in the liver of mice with MASLD, suggesting that SPA may contribute to the pathogenic mechanisms implicated in the development of MASLD. The reduced lipid accumulation in SPA-deficient mice observed in this study implies that SPA increases hepatic steatosis, compromising hepatocyte membrane integrity, allowing fluid influx and loss of cell shape with resultant swelling, and rounding up of hepatocytes (hepatocyte ballooning). These processes eventually cause significant damage to the cytoskeleton and promote hepatocyte degeneration^39^. The increased lobular inflammation can result from excessive production of cell adhesion molecules by the damaged hepatocytes, which attract immune cells and subsequent infiltration in response to hepatocyte degeneration. Previous studies have shown that macrophage recruitment via increased expression of monocyte chemoattractant protein 1 (MCP-1, also referred as CCl2) in hepatocytes enhanced hepatic steatosis and inflammation in alcoholic and nonalcoholic liver disease^40,41^.

Another major factor contributing to MASLD is triglyceride accumulation in hepatocytes, which occurs due to imbalanced lipid input and lipid export^42^. The decreased liver TG and cholesterol in SPA deficient mice observed in this study suggest that SPA may promote lipid acquisition either by direct uptake of fatty acids from circulation or hepatic production of fatty acids from carbohydrates, with reduced lipid removal, thereby causing lipid accumulation in hepatocytes. Liver enzymes (ALT, AST, and ALP) act as catalysts that enhance critical biochemical reactions (such as amino transfer) but leak into circulation due to membrane damage in hepatocytes. Thus, increased serum concentration of these enzymes has been considered a biomarker of liver injury^43^. Elevated serum AST has been correlated with fibrosis in patients with MASLD, while increased ALT concentrations have been reported in fatty liver due to hepatic insulin resistance^44^. The levels of these enzymes were significantly reduced in W-D-fed SPA-deficient mice, suggesting that SPA may aggravate liver injury in MASLD.

It has been established that fatty acid uptake, de novo lipogenesis, and fatty acid oxidation play critical roles in lipid homeostasis. Fatty acid uptake via PPARγ and CD36 and lipogenic genes (FASn and SCD1) increase lipid import in hepatocytes while major genes regulating fatty acid oxidation (CPT1a and PPARα) promote lipid output^45^. Results from this study suggest that SPA affects hepatic fatty acid accumulation by upregulating the expression of PPARγ and CD36 that enhance the uptake of fatty acid into the liver cells because they were both significantly reduced in livers of SPA^−/−^ mice. Consistent with other reports, PPARγ is a positive regulator of CD36, and upregulation of this pathway (PPARγ-CD36) has been shown to mediate hepatic lipid uptake, especially during a fatty diet^46,47^. However, CPT1a, PPARα, FASn, and SCD1 were not affected in SPA^−/−^ mice compared to WT mice fed W-D, suggesting that SPA is not involved in de novo lipogenesis and fatty acid oxidation.

Inflammatory cell infiltration, macrophage activation, and severity of fibrotic lesions are characteristic features that differentiate MASLD from simple steatosis^48^. Lipotoxicity facilitates macrophage polarization with a resultant increase in pro-inflammatory macrophage and secretion of various cytokines that promote the development of steatohepatitis and inflammatory response in the hepatocytes^49^. Steatosis can also increase the expression of cell adhesion molecules, such as VCAM-1, by the liver sinusoidal endothelial cells, thereby recruiting monocytes into the liver and promoting the pathogenesis of MASLD^50^. Thus, liver inflammation is another major hit in the spectrum of MASLD. Previous studies have shown that VCAM-1 is a surface protein that may link lipotoxicity with endothelial dysfunction in MASLD by promoting cellular adhesion and infiltration of monocytes^34^. Thus, immune cell infiltration might also be due to increased expression of VCAM-1 in the livers resulting from the lipotoxicity. In addition, the upregulation of inflammatory mediators in WT mice fed W-D but downregulated in SPA-deficient mice further implies that SPA may promote liver inflammation to exacerbate the pathogenesis of MASLD.

Stellate cells account for approximately 5–8% of the total number of cells in the liver, and these cells are always in a quiescent state. Activation of stellate cells promotes extracellular matrix expansion and accumulation, ultimately resulting in fibrosis, which may progress to cirrhosis and liver failure^51^. In MASLD, lipotoxicity and inflammatory molecules activate Kupffer cells, which in turn promote stellate cell phenotypic switch from quiescent fibroblasts to myo-fibroblasts which secrete fibrogenic factors and cause collagen deposition^52^. Thus, fibrosis has been recognized as a critical pathological feature in MASLD, defining a severe and advanced form of liver injury. This study demonstrates that SPA deficiency attenuated stellate cell activation in W-D fed mice, which may have resulted from the reduced inflammation observed in SPA^−/−^ mice fed W-D. Consequently, the reduced mRNA expression of fibrogenic genes (Col1a1 and TGFβ1) and collagen deposition observed in the SPA-deficient mice suggests that SPA promotes stellate cell synthetic phenotype in MASLD. These data indicate that SPA contributes to MASLD-related fibrosis by activating hepatic stellate cells.

In conclusion, this study used W-D feeding as an animal model of MASLD, which has been reported to mimic a spectrum of features like human MASLD, including hepatic steatosis, liver inflammation, and fibrosis^53^. Obesity and insulin resistance are commonly reported in human MASLD, but these were not observed in W-D fed mice. However, results from this study provide evidence that SPA promotes lipid accumulation, hepatic inflammation, and fibrosis by increasing fatty acid uptake and activating stellate cells in MASLD, suggesting that SPA inhibition may be a potential therapeutic strategy to treat MASLD.

### Supplementary Information

Supplementary Information.

## Data Availability

All relevant data will be made available from the corresponding author upon reasonable request.
