# Supplementary material for: Surfactant protein A promotes western diet-induced hepatic steatosis and fibrosis in mice
Source: Sci Rep. 2024 Mar 29;14:7464. doi: 10.1038/s41598-024-58291-5 (PMC10980756; doi:10.1038/s41598-024-58291-5)
Supplement: Supplementary file 1 — Supplementary Information. [file 41598_2024_58291_MOESM1_ESM.pdf]

Surfactant protein A promotes western diet-induced hepatic steatosis and fibrosis in mice

Ayobami Dare[1], Skylar D. King[1], Shi-You Chen[1,2*]

[1]Department of Surgery, University of Missouri School of Medicine, Columbia, MO 65212;
[2]The Research Service, Harry S. Truman Memorial Veterans Hospital, Columbia, MO, 65201

*** Corresponding Author:**

Shi-You Chen, Ph.D.

University of Missouri School of Medicine

Department of Surgery

1 Hospital Drive

Columbia, MO 65212

Tel: (573) 884-0371

Email: scqvd@missouri.edu

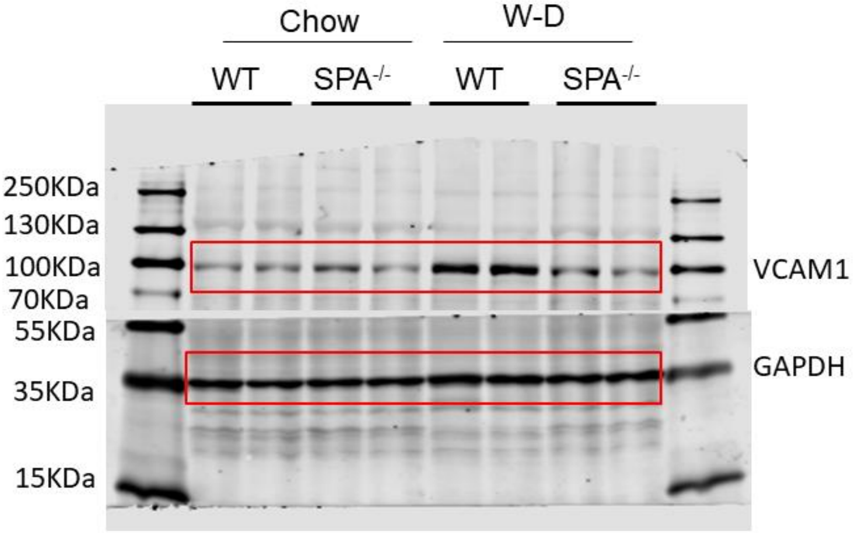

Figure-1: Western blotting for VCAM1 and GAPDH (loading control) in WT or SPA-/- mice fed Chow or W-D. The red rectangle indicates the cropped area displayed in the main figures.

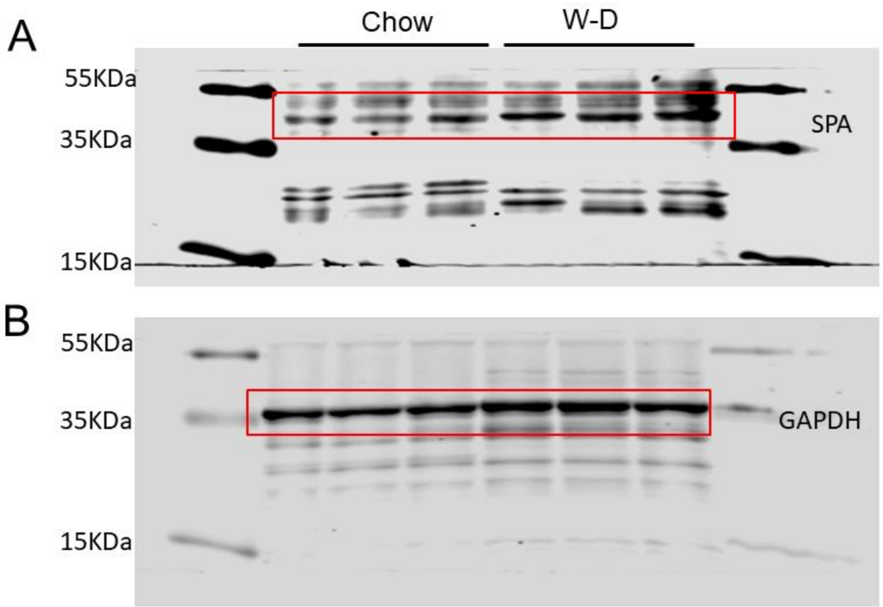

Figure 2: Westernblotting for SPA (a) and GAPDH (b) in WT mice fed Chow or W-D. The red rectangle indicates the cropped area displayed in the main figures.
